# *Ex Vivo* and *In Vivo* Biocompatibility Assessment (Blood and Tissue) of Three-Dimensional Bacterial Nanocellulose Biomaterials for Soft Tissue Implants

**DOI:** 10.1038/s41598-019-46918-x

**Published:** 2019-07-22

**Authors:** M. Osorio, A. Cañas, J. Puerta, L. Díaz, T. Naranjo, I. Ortiz, C. Castro

**Affiliations:** 1 School of Engineering, Universidad Pontificia Bolivariana, Circular 1 # 70-01, Medellín, Colombia; 2 School of Health Sciences, Universidad Pontificia Bolivariana, Calle 78 B # 72 A-109, Medellín, Colombia; 3 Medical and Experimental Mycology Group, Corporación para Investigaciones Biológicas, Carrera 72 A # 78 B-141, Medellín, Colombia

**Keywords:** Cell migration, Polysaccharides

## Abstract

Bacterial nanocellulose (BNC) is a promising biomedical material. However, the haemocompatibility (haemolysis and thrombogenicity) and acute and sub-chronic immune responses to three-dimensional (3D) BNC biomaterials have not been evaluated. Accordingly, this manuscript focused on the effect of 3D microporosity on BNC haemocompatibility and a comparison with 2D BNC architecture, followed by the evaluation of the immune response to 3D BNC. Blood *ex vivo* studies indicated that compared with other 2D and 3D BNC architectures, never-dried 2D BNC presented antihemolytic and antithrombogenic effects. Nevertheless, *in vivo* studies indicated that 3D BNC did not interfere with wound haemostasis and elicited a mild acute inflammatory response, not a foreign body or chronic inflammatory response. Moreover, compared with the polyethylene controls, the implant design with micropores *ca*. 60 µm in diameter showed a high level of collagen, neovascularization and low fibrosis. Cell/tissue infiltration increased to 91% after 12 weeks and was characterized by fibroblastic, capillary and extracellular matrix infiltration. Accordingly, 3D BNC biomaterials can be considered a potential implantable biomaterial for soft tissue augmentation or replacement.

## Introduction

Bacterial nanocellulose (BNC) is composed of linked units of β-1,4-D-(+)-glucose^1^. BNC is produced by strains such as *Acetobacter, Sarcina, Gluconacetobacter*, and *Komagataiebacter*^2–4^. As a natural nanoscale biomaterial, BNC possesses different characteristics from other cellulose types, including its morphology (collagen biomimicry), biocompatibility, and lack of toxicity^5^, which have been thoroughly explored in biomedical areas, mainly as a two-dimensional (2D) biomaterial^6–10^. Use of a three-dimensional (3D) biomaterial for cell regenerative and tissue engineering approaches allows cell infiltration to the inner areas of the material^11,12^. The natural pores in BNC are small (lower than 1 µm in an open mesh^13^), inhibiting cell infiltration (cells are generally larger than 20–40 µm^14^); thus, BNC is inherently a 2D biomaterial. The applications of 2D BNC wound dressings, heart valves, dura-matter membranes, blood vessels, and dermal drug delivery systems, among others, have been explored^6–10^.

Recently, some 3D BNC biomaterials have been reported in the literature. Bioprinting is one of the most commonly used approaches. For instance, Markstedt *et al*.^15^ produced an ear-shaped BNC/alginate cartilage using bioprinting^15^, and Martinez Ávila *et al*.^16^ 3D bioprinted human chondrocyte-laden nanocellulose hydrogels for patient-specific auricular cartilage regeneration^16^, both of which were seen as promising approaches for the regeneration of auricular cartilage in *in vitro* evaluations^15,16^. Nevertheless, there are also other strategies, such as the inclusion of porogenic agents^17^ and freeze-drying^5,18^. For instance, Krontiras *et al*.^18^ used 3D microporous BNC generated via nanofibril disintegration and freeze-drying to differentiate *in vitro* adipogenic stem cells^18^, Osorio *et al*.^17^ developed a novel methodology for leaching paraffin microspheres to generate 3D interconnected micropores in BNC during the fermentation of bacteria, and Porto *et al*.^19^ reported a microarchitectural and radiological study of cocoon-like 3D BNC generated using agitated culture conditions^19^.

3D implants for soft tissue promote neovascularization and improved tissue integration^20–27^ and provide the appropriate microenvironment for the regeneration of tissues and organs by acting as a template for tissue formation^27^. Consequently, blood and immune reactions are important for addressing the performance of future 3D BNC implants^28^. The blood and immune systems are complex systems that feature chemical reactions in cascades. However, for implantable devices, haemolysis, clotting time and acute and sub-chronic inflammatory reactions need to be assessed^28^, especially for non-resorbable biomaterials made of BNC that are expected to be in contact with the human body for a lifetime^29^.

There are reports regarding the assessment of BNC in *in vivo* models. However, most of them are focused on 2D BNC. For instance, Malm *et al*.^10^ evaluated BNC as a small-calibre blood vessel in a sheep model^10^; however, they evaluated only the interaction of 2D BNC with blood, but they did not investigate the immune reaction to the biomaterial in depth. In addition, mice have been implanted subcutaneously with BNC^29–31^, but those experiments were carried out using 2D biomaterials, systems that scarcely allow cell infiltration. Additionally, pigs have been implanted with BNC but as a meniscus implant composed of 2D BNC for orthopaedic applications^32^. Ávila *et al*.^33^ evaluated a 3D BNC/alginate biomaterial for cartilage applications and assessed them *in vivo*^33^. However, they evaluated a mixture rather than pure nanocellulose, and they did not evaluate the influence of the pore diameter on tissue infiltration.

Accordingly, the aim of this work is to evaluate the effect of the pore diameter on the *ex vivo* haemocompatibility (haemolysis and clotting time) of and short- and long-term *in vivo* implantation response to microporous 3D BNC biomaterials. Furthermore, this manuscript assesses the haemocompatibility of 2D BNC for comparison purposes because although some applications of blood-contacting biomedical devices comprising 2D BNC were found in the literature^5,8,34^, there is no assessment of the haemocompatibility of those biomaterials.

## Results

### Biomaterial microstructure

Table 1 presents the nomenclature of the biomaterials tested in this paper, along with the 3D diameter or the drying state.

**Table 1:** Biomaterial nomenclature.

BNC	Microsphere’s diameter (µm)/Drying state	Developed Biomaterial
3D*	280.7 (88.7)	BNC-MD
	136.3 (49.5)	BNC-SM
	61.2 (12.0)	BNC-XS
2D	Never dried	BNC-ND
	Oven dried	BNC-OD
	Freeze dried	BNC-FD

*All 3D BNC biomaterials are in never dried state.

The diameter was measured using light microscopy images at 100X and ImageJ. In brackets, the standard deviation from the measurement of 100 microspheres is presented.

**Figure 1:**
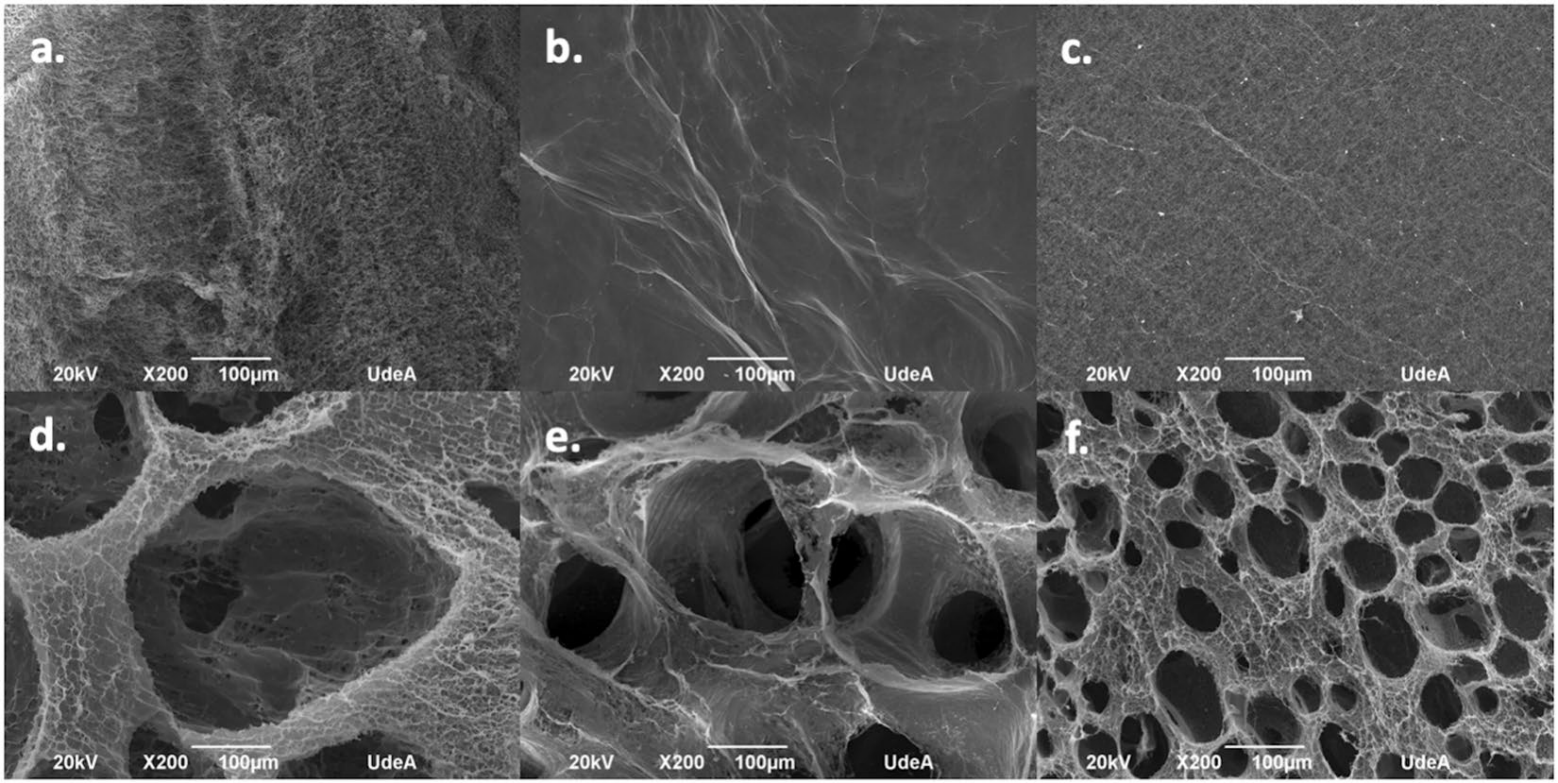
2D and 3D BNC biomaterial microstructures. The scale bar represents 100 µm. (**a**) BNC-ND, (**b**) BNC-OD, (**c**) BNC-FD, (**d**) BNC-MD, **(e**) BNC-SM, (**f**) BNC-XS.

The topography of 3D and 2D BNC biomaterial microstructures fabricated using *Komagataeibacter medellinensis* is presented in Fig. 1. In the 2D biomaterials, BNC-ND (Fig. 1a.) has a rough surface due to the presence of free entangled BNC nanoribbons; however, in its natural state, the surface is lubricated because of the liquid content of its hydrogel state^35^. BNC-OD (Fig. 1b) has a smooth surface because of the collapse of the BNC nanoribbons during oven drying due the irreversible strong hydrogen bonding between the nanoribbons that generates a highly packed microstructure^36^; BNC-FD (Fig. 1c) presents a rough surface, but it is not lubricated because the drying process removes the liquid content of the biomaterial.

The 3D BNC biomaterials present an interconnected network of pores of various diameters according to Table 1. In these materials, the pore walls exhibit a rough and lubricated surface because they were made using never-dried BNC.

### Hemocompatibility

Hemolysis and clotting times were used to assess the haemocompatibility of the BNC-based biomaterials. These tests are based on the contact of erythrocytes and whole blood with the biomaterial surface. In the following sections, the results of these experiments are described.

For haemolysis, high-density polyethylene (HDPE) and plastisol were used as negative and positive controls, respectively, as both of them are recommended by the ASTM standard protocol and ISO 10993-12;^37,38^ furthermore, HDPE was used as a control in the thrombogenicity test because it has demonstrated superior blood biocompatibility^39^. For haemolysis, HDPE is inert and hydrophobic and has sliding properties, which reduce its contact with red blood cells^39–41^, avoiding their lysis; for thrombogenicity, among the materials tested, HDPE induces the lowest generation of factor XII and kallikrein, reducing the contact activation of blood and intrinsic coagulation^42^.

Plastisol is a haemolytic material because it gradually releases plasticizers such as polyethylene glycol monoalkyl ethers^43^, which destroy the cell membrane through a detergent effect^44^.

#### Haemolysis

The first step in assessing haemocompatibility is to assess haemolysis. This evaluation was divided to assess both the effect of drying 2D BNC and the effect of 3D microporosity in BNC biomaterials.

**Figure 2:**
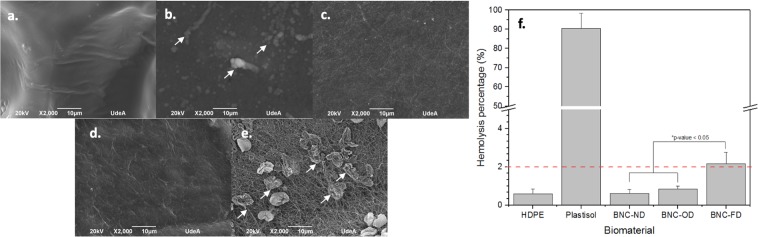
Effect of drying of 2D BNC biomaterials on haemolysis. SEM micrographs after the haemolysis test: (**a**) high-density polyethylene, (**b**) plastisol, (**c**) BNC-ND, (**e**) BNC-OD, (**e**) BNC-FD; the white arrows indicate lysed red blood cells. (**f**) Haemolysis percentage, where the dashed red line is the cut-off for haemolytic behaviour.

Effect of drying of 2D BNC: Figure 2 presents the results for the effect of drying on haemolysis.

BNC-FD (freeze-dried) exhibited hemolytic behaviour (see Fig. 2f), which could be related to its microstructure, as freeze-drying removes the liquid from the material, leaving a sponge composed of BNC nanoribbons. When red cells are added to the biomaterial surface, the biomaterial easily adsorbs the liquid and generates a strong interaction (friction) between the red cells and BNC nanoribbons, causing friction that can weaken the cell membrane (see Fig. 2e arrows) and induce red blood cell lysis, *i.e*. haemolysis. The morphology of red blood cells is a smooth biconcave disc^45^ that was disintegrated upon contact with BNC-FD and plastisol. In the case of never-dried BNC (BNC-ND), this interaction is reduced by the presence of liquid (never-dried BNC can hold up to *ca*. 95–99 wt. % of liquid^46,47^) that lubricates the biomaterial surface, reducing the haemolysis percentage to acceptable values compared with that induced by HDPE (negative control). Although BNC-OD is free of liquid, this material does not induce any haemolytic behaviour because of its highly packed microstructure, which generates a smooth surface and reduces the friction between red blood cells and the biomaterial.

**Figure 3:**
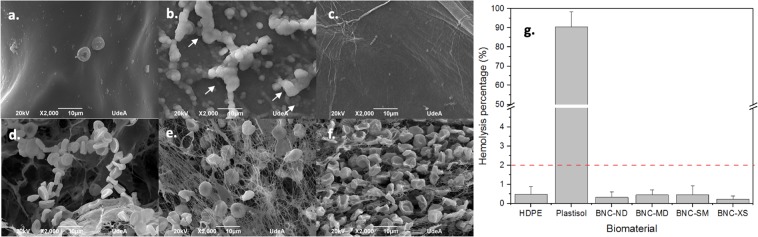
Effect of the 3D microporosity in BNC on haemolysis. SEM micrographs after the haemolysis test: (**a**) high-density polyethylene, (**b**) plastisol, **(c**) BNC-ND, **(d**) BNC-MD, (**e**) BNC-SM, (**f**) BNC-XS; the white arrows indicate lysed red blood cells. (**g**) Haemolysis percentage, where the dashed red line is the cut-off for haemolytic behaviour.

Effect of 3D microporosity in BNC: Regarding the 3D BNC biomaterials (Fig. 3), it was shown that none of the 3D architectures induced haemolytic behaviour; furthermore, there were no statistically significant differences between the biomaterials and the negative control. However, it was interesting to find that the materials with extra small porosities (*ca*. 60 µm) entrapped a high number of red cells; nevertheless, these cells exhibit their natural morphologies (smooth biconcave discs^45^) and have stable cell membranes. Accordingly, in comparison with non-porous BNC biomaterials, 3D BNC biomaterials with porous networks induce higher interactions with red blood cells; however, because of the presence of liquid that lubricates their nanoribbon surface, these biomaterials do not cause haemolysis.

#### Thrombogenicity (clotting time)

Effect of drying of 2D BNC: The activation of the extrinsic coagulation pathway by the biomaterials was followed using SEM micrographs and clotting time profiles. The coagulation is due to the adsorption of plasma proteins on the biomaterial surface triggering the clotting cascade^48^. The following figure shows the effect of drying on 2D BNC thrombogenicity.

**Figure 4:**
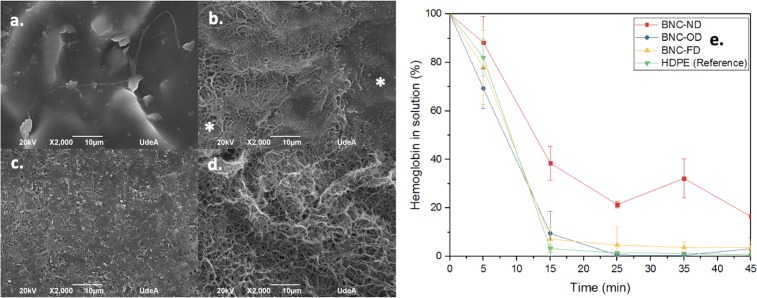
Thrombogenicity assessment and effect of drying. SEM micrographs of the adsorption of plasma proteins: (**a**) HDPE (reference), (**b**) BNC-ND, (**c**) BNC-OD, (**d**) BNC-FD. (**e**) Clotting time profiles of biomaterials and the reference. The scale bar length is 10 µm, and *indicates surfaces not covered by plasma proteins.

Plasma proteins, including fibrin, are absorbed on all biomaterials, even on HDPE, which is considered the least thrombogenic biomaterial in the literature^49^. According to Fig. 4e, at 15 minutes, there was complete clot formation for HDPE, BNC-OD and BNC-FD, but surprisingly, BNC-ND did not form a full clot even after 45 minutes, behaviour that can be attributed to the presence of water that reduced the deposition of plasma proteins, preventing clotting formation. These results were also corroborated by the SEM images. A packed plasma protein layer can be observed on HDPE and BNC-OD (Fig. 4a,c), possibly indicating that factor XIIIa was activated and cross-linked the fibrin fibres^50^. For BNC-FD, a homogeneous mesh of plasma proteins was deposited on the BNC surface (Fig. 4d). However, in the case of BNC-ND (Fig. 4b), there are still some areas not covered by the plasma proteins; *i.e*., the clotting cascade is not fully activated by BNC-ND, as the plasma proteins were not completely deposited.

**Figure 5:**
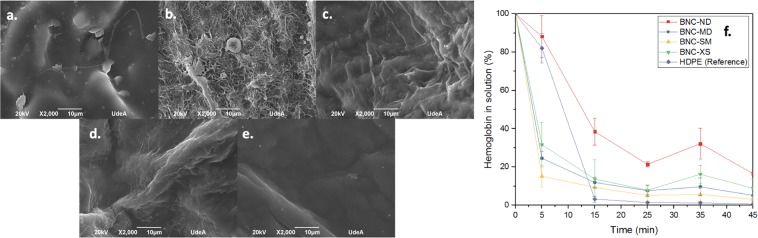
Thrombogenicity assessment and effect of 3D microporosity. SEM micrographs of plasma protein adsorption: (**a**) HDPE (reference), (**b**) BNC-ND, (**c**) BNC-MD, (**d**) BNC-SM, (**e**) BNC-XS. (**f**) Clotting time profiles of biomaterials and the reference. The scale bar length is 10 µm.

Effect of 3D microporosity in BNC: Compared to BNC-ND (Fig. 4b), the 3D BNC biomaterials (see Fig. 5) in which 3D micropores were incorporated display accelerated plasma protein adsorption, showing behaviour similar to that of the HDPE control. The plasma proteins were observed to be highly packed on the biomaterial surface because full clot formation was complete at 15 minutes for HDPE, BNC-MD, BNC-SM, and BNC-XS.

3D micropores facilitate the adsorption of plasma proteins and then enable the activation of factor XIIIa, as described for other biomaterials elsewhere in the literature^51–53^, where a higher surface area at the microscale promoted plasma protein adsorption and deposition^54^. However, despite the reduction in clotting time, the biomaterials still behave similar to the HDPE reference.

### *In vivo* assessment of the immune response to 3D BNC biomaterials

#### Macroscopic analysis

**Figure 6:**
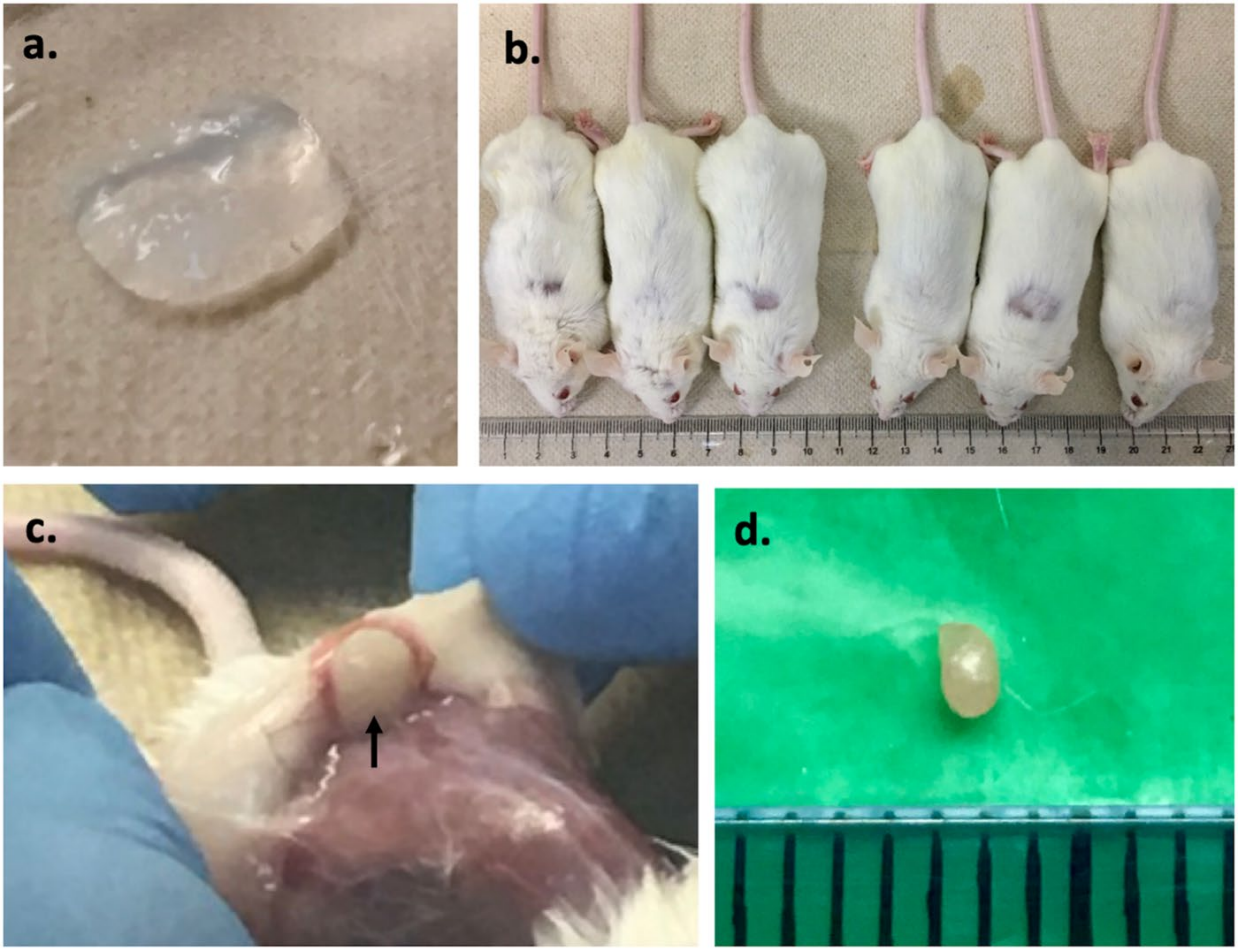
Macroscopic evaluation of the implantation and explantation of the biomaterials. (**a**) Biomaterial appearance before implantation; (**b**) mice at explantation time; (**c**) biomaterial appearance during explantation, where the black arrow indicates the biomaterial; (**d**) lymphatic ganglion.

Figure 6 shows the gross inspection of the implantation and explantation processes of the biomaterials. The biomaterials appear as translucent hydrogels (see Fig. 6a). During the test, none of the animals showed signs of discomfort, pain or disease at any time. The mice showed active behaviour, and their eyes remained active. After the first week, the wound had healed, and new hair growth was observed, as shown in Fig. 6b. At explantation, the biomaterials maintained their shape, there was no sign of dehydration (see the biomaterial indicated by the arrow in Fig. 6c), and no purulent inflammation or adverse tissue reactions were observed (necrosis, bruising or edema). All samples showed encapsulation by fibrovascular tissue. Macroscopically, all the biomaterials were enveloped in thin tissue capsules.

Analysing the lymphatic ganglion of all the mice per time revealed that the appearance was similar to that of a normal creamy kidney-shaped ganglion of 1 mm diameter (Fig. 6d) without any sign of necrosis, extended inflammation or edema.

#### Microscopic analysis

The quality of the cell/tissue infiltrate was investigated using histology, and the results are presented in Table 2. Latex and HDPE were used as positive and negative controls, respectively.

**Table 2:** Cell response to each biomaterial (latex, HDPE, BNC-ND, BNC-MD, BNC-SM, and BNC-XS) at different times (1, 4, 8 and 12 weeks).

Cell type/response Scores								
Biomaterial	Latex^+^							
Weeks	Polymorphonuclear cells	Lymphocytes	Plasma Cells	Macrophages	Neovascularization	Fibrosis	Collagen	Elastin
1	2	1	1	2	1	1	2	0
4	2	0	0	0	2	3	4	1
8	1	0	0	0	1	1	2	2
12	2	3	0	0	0	3	3	2
**Biomaterial**	**HDPE** ^**+**^							
**Weeks**	**Polymorphonuclear cells**	**Lymphocytes**	**Plasma Cells**	**Macrophages**	**Neovascularization**	**Fibrosis**	**Collagen**	**Elastin**
1	3	0	0	1	0	0	1	0
4	1	0	0	0	2	1	1	1
8	0	0	0	0	0	1	2	0
12	0	0	0	0	0	2	2	2
**Biomaterial**	**BNC-ND** ^**++**^							
**Weeks**	**Polymorphonuclear cells**	**Lymphocytes**	**Plasma Cells**	**Macrophages**	**Neovascularization**	**Fibrosis**	**Collagen**	**Elastin**
1	2	0	0	1	0	0	1	0
4	0	0	0	0	1	1	2	1
8	0	0	0	0	0	0	2	2
12	0	0	0	0	0	1	1	1
**Biomaterial**	**BNC-MD** ^**++**^							
**Weeks**	**Polymorphonuclear cells**	**Lymphocytes**	**Plasma Cells**	**Macrophages**	**Neovascularization**	**Fibrosis**	**Collagen**	**Elastin**
1	1	0	0	1	0	0	1	1
4	0	0	0	0	0	1	2	2
8	1	0	0	0	1	0	3	2
12	0	0	0	0	0	0	2	1
**Biomaterial**	**BNC-SM** ^**++**^							
**Weeks**	**Polymorphonuclear cells**	**Lymphocytes**	**Plasma Cells**	**Macrophages**	**Neovascularization**	**Fibrosis**	**Collagen**	**Elastin**
1	1	1	0	0	0	0	2	0
4	2	1	0	0	0	0	2	1
8	1	0	0	0	1	0	2	1
12	0	0	0	0	1	0	1	1
**Biomaterial**	**BNC-XS** ^**++**^							
**Weeks**	**Polymorphonuclear cells**	**Lymphocytes**	**Plasma Cells**	**Macrophages**	**Neovascularization**	**Fibrosis**	**Collagen**	**Elastin**
1	1	1	0	1	0	0	2	0
4	0	0	0	0	2	0	2	2
8	1	0	0	0	2	0	3	2
12	0	0	0	0	1	0	3	2

^+^Cell/tissue response around the biomaterial; ^++^Cell/tissue response around and inside the biomaterial. Five fields at 40X were analysed per biomaterial, and the results were averaged to assess the global score.

**Figure 7:**
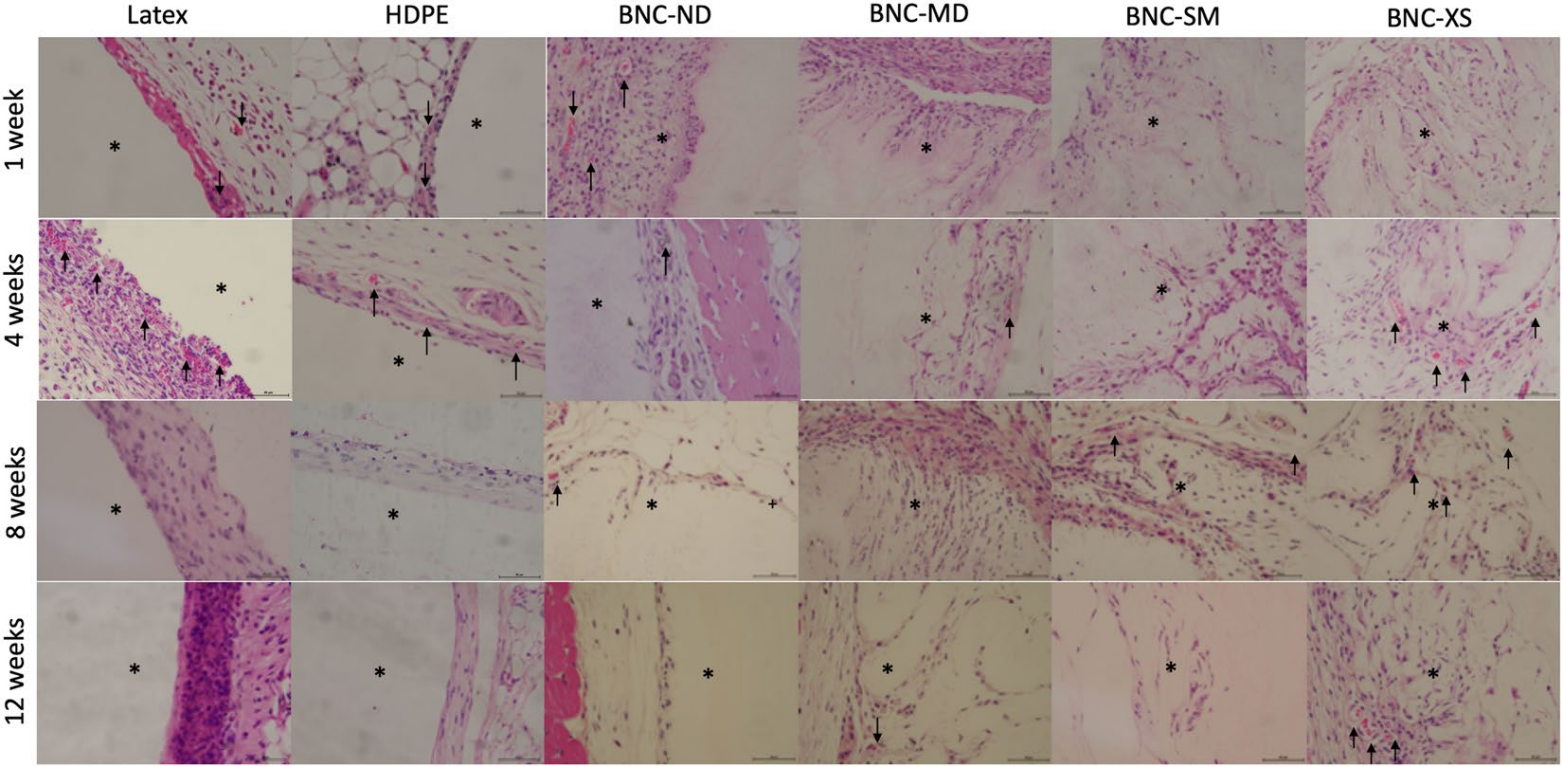
Haematoxylin-eosin staining of each biomaterial at different times, where purple denotes the nucleus and pink denotes the cytoplasm. Images were recorded at 40X, and the scale bar represents 50 µm. *Indicates the presence of the biomaterial, and neovascularization is denoted by black arrows.

**Figure 8:**
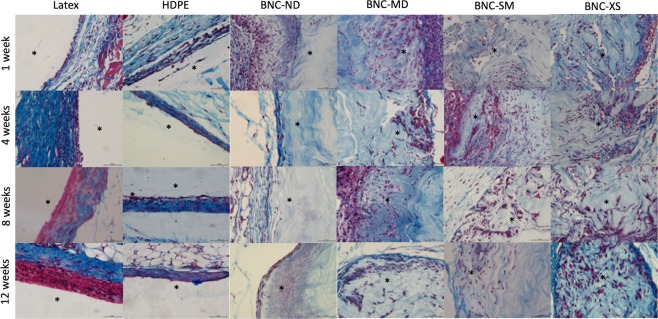
Masson’s trichrome staining of each biomaterial at different times. Red denotes keratin and muscle fibres, blue denotes collagen, light red denotes the cytoplasm, and black denotes the cell nuclei. Images were recorded at 40X, and the scale bar represents 50 µm. *Indicates the presence of the biomaterial.

**Figure 9:**
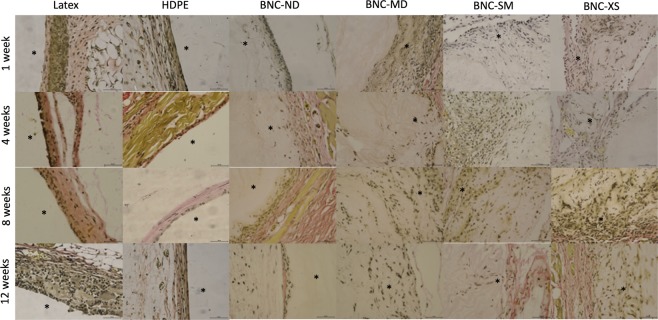
Verhoeff-van Gieson staining of each biomaterial at different times. Elastic fibres and cell nuclei are stained black, collagen fibres are stained pink, and other tissue elements, including the cytoplasm, are stained yellow. Images were recorded at 40X, and the scale bar represents 50 µm. *Indicates the presence of the biomaterial.

The HDPE negative control elicited an acute inflammatory reaction (1 week) characterized by heavy polymorphonuclear cells, mild lymphocytes and minimal presence of macrophages in the injury site. At week 4, the inflammation was minimized, and then the injury was resolved, as shown by the haematoxylin-eosin images in Fig. 7. Regarding neovascularization, some buds were presented around HDPE during the first four weeks. Figures 8 and 9 present Masson’s trichrome and Verhoeff-van Gieson staining images, respectively, which are used to reveal the deposition of extracellular matrix proteins, collagen and elastin, as well as to elucidate the presence of fibrosis. Collagen and elastin were present around HDPE; moreover, it can be seen that the fibres align with the biomaterial surface, generating a narrow to moderately thick fibrotic band, which is common behaviour for most implantable biomaterials^55^.

Latex, as a positive control, is an irritating and toxic material^56^. During the *in vivo* test, this material induced the maximum inflammation response during the acute response stage, and lymphocytes were detected up to the 12^th^ week. Furthermore, the inflammation did not decrease over time, and in the last week, there was a thick band of lymphocytes and fibrotic tissue present around the biomaterial, which is a sign of chronic inflammation^57^; nevertheless, there was no evidence of traumatic necrosis.

Regarding the BNC biomaterials, the non-porous biomaterial (BNC-ND) elicited mild acute inflammation characterized by the presence of polymorphonuclear leukocytes in the first week. At the 4^th^ week, there were no inflammatory cells; *i.e*., the immune reaction was resolved on average. Similar to HDPE, neovascularization occurred around the biomaterial and was evident at the 4^th^ week. According to Table 2, a narrow layer of collagen was deposited around the biomaterial, scoring up to 1 at the 12^th^ week, while the replicates of the negative control scored up to 2, indicating lower fibrosis (see also Figs 8 and 9).

In comparison with HDPE and BNC-ND, the 3D porous BNC biomaterials (BNC-MD, BNC-SM and BNC-XS) showed a lower immune response at the first week because the presence of 3D micropores allowed the immune cells to move inside the biomaterial rather than concentrate at the edges; thus, the cells were better distributed within the biomaterial. However, the inflammation was resolved after the 8^th^ week, while in HDPE and BNC-ND, the inflammation was resolved after the 4^th^ week, which is related to the higher surface area that keeps leukocytes active while they are infiltrating inside BNC. Nevertheless, these cell infiltrates were characterized by the predominant presence of polymorphonuclear leukocytes; *i.e*., there was no evidence of chronic inflammation. Furthermore, the infiltration of polymorphonuclear leukocytes is advantageous because they release cytokines and chemoattractants for fibroblasts, the cells that restore the extracellular matrix^58^. For this reason, more collagen and elastin was found for the 3D BNC biomaterials than HDPE and BNC-ND, and because of the better distribution of collagen within the biomaterial, the fibrosis was, on average, absent or narrow. Neovascularization was also observed in the porous biomaterials, especially in BNC-XS, attaining scores up to 2, *i.e*., groups of 4–7 capillaries with supporting fibroblastic structures (see Fig. 7, last row), some of which were retracted at the 12^th^ week as part of the natural process of remodelling^59^.

**Figure 10:**
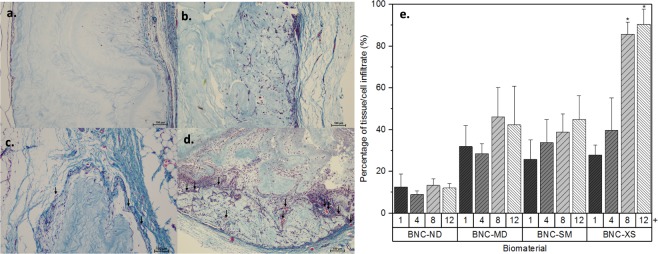
Tissue/cell infiltrate in BNC biomaterials, Masson’s trichrome staining images recorded at 10X on the 12^th^ week. The scale bar represents 100 µm, and the arrows indicate the presence of capillary buds. (**a**) BNC-ND; (**b**) BNC-MD; (**c**) BNC-SM; (**d**) BNC-XS; (**e**) percentage of tissue/cell infiltrate in the biomaterials; + time in weeks, neovascularization (black arrows), *groups that are significantly different (p-value less than 0.05).

The main goal of using 3D scaffolds is to allow high cell/tissue infiltration. Figure 10 shows the average cell infiltration in the BNC biomaterials. The controls (latex and HDPE) did not present cell/tissue infiltration (see Figs 7–9); for this reason, they were excluded in the following analysis.

2D BNC, *i.e*., BNC-ND, exhibits narrow tissue/cell infiltration, less than *ca*. 15%, as cells were not able to go further than 100 µm; similar behaviour was observed in previous reports^29–31^. Cells are thought to be able to push BNC nanoribbons and penetrate into the biomaterial. However, at the same time, cells are compressing the nanoribbons in front of them, and at 100 µm, the cells cannot go further. The inclusion of 3D micropores on BNC remarkably enhanced the tissue/cell ingrowth; moreover, biomaterials with porosities of 60 µm attained 90.53 ± 7.27% tissue/cell infiltration after 12 weeks. Biomaterials with microporosities of 136 µm and 281 µm in diameter attained a maximum infiltration of 40%. Accordingly, the surface area generated by small interconnected porosities has a stronger effect than large pore diameters. Furthermore, capillary vessels within BNC-XS can be observed (see arrows in Fig. 10d); these vessels are important for enhancing cell infiltration because they supply nutrients and oxygen to the cells, allowing their movement within the biomaterial^60,61^. In BNC-ND, BNC-MD or BNC-SM, no capillary vessels were present within the biomaterials, although some were found around the implant. Again, interconnectivity and surface area promote capillary infiltration because the pore network acts as a template for blood vessel ingrowth^61^.

## Discussion

The biocompatibility of implants and medical devices depends on the biological events occurring at the interfaces between the biomaterials and surrounding tissue^62^. Surface properties such as (1) surface free energy and wettability, (2) surface chemistry and functional groups, and (3) surface topography and roughness influence the response of the blood and immune systems to the biomaterial^62^. The first two properties depend on the chemical composition of the biomaterial, which is the same for all the bacterial nanocellulose 2D and 3D biomaterials evaluated in this study. For the third property, the inclusion of micropores and the drying process of 2D BNC influence the biomaterial topography and subsequently the blood and immune response.

The initial concern was about the effect of the drying process of 2D BNC on its haemocompatibility. The drying method generates three different 2D surfaces: a smooth surface, (BNC-OD), a rough surface (BNC-FD) and a lubricated surface (BNC-ND). The results showed that a lubricated surface has better performance than a rough surface, which is similar to behaviour found for other systems, such as poly(vinylidene fluoride), where a water layer on a highly hydrated surface repelled plasma proteins and erythrocytes and generates an antithrombogenic and antihemolytic surface^63,64^. Moreover, other studies noted that mechanical friction causes haemolysis when erythrocytes are forced to pass over a rough surface^65,66^. A smooth BNC surface prevents erythrocyte lysis but fails to prevent the activation of the coagulation cascade, as BNC in the dry state features more hydrogen donor groups to interact with plasma proteins^67^, a process that will facilitate their absorption.

The inclusion of 3D microporosities had no negative effect on haemolysis, and despite promoting the activation of the coagulation cascade in comparison with non-porous BNC (greater surface area, more hydrogen bond donor groups), it was found that the biomaterials are not inferior to the HDPE reference. Moreover, there was no evidence of clots around or inside the biomaterials at the first week in the *in vivo* studies (see Fig. 7); *i.e*., the biomaterials do not induce thrombosis and do not interfere with wound haemostasis^68^.

During the first week, the BNC biomaterials showed minimal to mild inflammation, and at the fourth week, there was evidence of fibroblasts (fusiform cells in Fig. 7) and blood vessels either around and inside the biomaterial as a consequence of the beginning of the proliferation phase in the healing process^59,69^. In this week, the score of polymorphonuclear cells was reduced, but they were still present until the 8^th^ week. The BNC implants in the present experiment did not elicit a foreign body reaction. Only a thin capsule was formed over time for the non-porous biomaterial (BNC-ND); for the 3D porous biomaterials, this capsule was thinner or not appreciable because cells infiltrate into the biomaterial rather than stay at the edges. Analysing cell infiltration revealed that the maximum percentage was observed at the 8^th^ week, after which the percentage was stable because the injury entered the remodelling phase^59^, causing the infiltration process to slow down. Among the biomaterials tested, BNC-XS exhibited the highest cell/tissue infiltration after the 8^th^ week.

Several researchers have been interested in finding the most suitable pore size for a 3D porous biomaterial. The answer depends on the tissue that the biomaterial is intended to replace; for instance, for bone regeneration researchers such as Baksh *et al*.^70^, Murphy *et al*. (2010) and Seyednejad *et al*.^71^, it was found that pore diameters above 300 µm are advantageous for bone migration^70–72^. For cartilaginous extracellular matrix, Feldmann *et al*.^73^ found no difference in synthesis between materials with porosities of 150–300 µm and 300–500 µm^73^. For soft tissue, Rnjak-Kovacina *et al*.^74^ found higher fibroblast proliferation in porous biomaterials of 11.7 µm diameter^74^. According to the results of this paper, porosities of *ca*. 60 µm provide better performance for soft tissue, as they allow increased cell and tissue infiltration and better extracellular matrix deposition (collagen and elastin), indicating that small interconnected pores are more relevant than larger pores for this application.

Pore size is also beneficial for blood vessel migration, as it facilitates nutrient and oxygen transport that may enhance the overall regeneration process^60,75,76^. Porosities of *ca*. 60 µm are also advantageous for blood vessel infiltration: higher numbers of blood vessel buds infiltrate up to 400 µm inside the biomaterial because of the high interconnectivity and surface area of the material acting as a template for capillary ingrowth^61^.

To conclude this paper, after evaluating BNC *ex vivo* and *in vivo*, it can be asserted that compared to dried 2D BNC, the hydrogel form of this biomaterial provides advantages in terms of haemocompatibility. Regarding the immune response to the 3D BNC biomaterial, there were no adverse responses, such as foreign body reactions, necrosis or chronic inflammation. Instead, it was found that biomaterials with porosities *ca*. 60 μm allowed high cell/tissue migration, excellent deposition of collagen and elastin, and superior fibrotic tissue distribution. Moreover, this pore diameter was found to be advantageous over larger pores of *ca*. 136 or 280 µm.

All the above results will facilitate the use of 3D BNC as an implantable biomaterial for soft tissue applications, for instance, as an augmentation or replacement material. Future works will focus on the inclusion of additional 3D structures, such as microconduits that can be used as support for peripheral capillary vessels, arterioles and venules and on the scaling of this technology to larger sizes that will allow the growth of organs and tissues of importance to the human body.

## Methods

### BNC biomaterial production

BNC was produced using Hestrin-Schramm culture media described elsewhere^5^. The medium was adjusted to pH = 3.6 with citric acid and then sterilized in an autoclave at 15 psi for 15 min. Two millilitres of culture medium was inoculated with *Komagataeibacter medellinensis* at 0.5 McFarland and distributed in a twelve-well polystyrene plate. Fermentation was carried out under static conditions for 7 days at 28 °C. BNC biomaterials were purified with 5 wt % KOH solution at room temperature for 14 h followed by continuous rinsing with phosphate-buffered saline (PBS) until pH *ca*. 7.4. The resultant material was sterilized in autoclave (121 °C for 15 min) for later use.

#### 2D BNC biomaterials

To investigate the effect of drying on biomaterial haemocompatibility, three 2D BNC biomaterials were tested in this work: never-dried BNC (BNC-ND), oven-dried BNC (BNC-OD) and freeze-dried BNC (BNC-FD). BNC-ND was produced as explained in 2.1. The BNC-OD biomaterials described in 2.1 were gravimetrically dried in a convention oven at 110 °C. BNC-FD was frozen at −196 °C using liquid nitrogen and dried for 72 h under 0.020 mbar vacuum pressure.

#### 3D BNC biomaterials

The development of 3D micropores in BNC involved the use of paraffin microspheres with the following particle diameters (see Table 1).

The spheres were produced according to the procedures described by Bäckdahl *et al*. and Zaborowska *et al*. Briefly, a solution of 0.5 wt. % polyvinyl alcohol (PVA) powder (Mw 114000) was allowed to dissolve in deionized water at 90 °C. The solution was heated to 70 °C and stirred at 1000–1200 rpm. Paraffin wax was allowed to melt on a hot plate. The melted paraffin was then poured slowly into the heated PVA solution to form particles^77,78^. The spheres were chilled using cold water, classified according to Table 1, and freeze-dried to remove the water.

Finally, to produce the 3D biomaterials, 1 ml of inoculated culture medium per well was distributed in 12-well polystyrene culture plates. The plates were left to generate an initial membrane for 3 days, and then 0.5 g of freeze-dried paraffin microspheres were added to each well, along with 200 µL of fresh culture media. Each 3 days, fresh culture medium was added until all the PMS was covered with BNC, and the fermentation was carried out at 28 °C under static conditions. After BNC covered the microspheres, the biomaterials were collected.

To expose the 3D structure, the paraffin microspheres were leached according to the detailed protocol described by Osorio *et al*. (2018). The method involves the use of hot water, ethanol and xylene^17^.

### *Ex vivo* haemocompatibility assays

#### Haemolysis

Haemolytic activity was tested according to the international standard ISO 10993-4^79^. The test is based on erythrocyte lysis induced by direct contact. The method is based on the release of haemoglobin by the lysis of red cells, which can be measured spectrophotometrically. Prior to the assay, a calibration curve was plotted using 6 concentrations of lyophilized human haemoglobin and Drabkin’s reagent (DR). The absorption was recorded at 540 nm, and the regression coefficient was R^2^ = 0.999.

Blood was aseptically collected from healthy adult donors in citrate tubes and then was centrifuged at 700–800 g for 30 min to collect erythrocyte-free plasma. The haemoglobin concentration in the plasma was measured by adding 0.5 ml of DR to 0.5 ml of plasma, incubating for 15 minutes at 37 °C and reading the absorption spectrophotometrically at 540 nm. The haemoglobin plasma concentration should be lower than 2 mg/ml to proceed with the test.

The haemoglobin concentration in whole blood (WB) was calculated by adding 20 µl of WB to 5 ml of DR, incubating and reading. Once the haemoglobin concentration in WB was measured, a proper dilution of WB was made according to Seyfert *et al*.^79^. Diluted blood was placed in contact with the materials at 37 °C for 3 h under shaking conditions to promote blood contact. After that time, the haemoglobin released into solution was measured. The experiments were performed in triplicate at three independent times. Controls were used according to ASTM F-756. The positive control was plastisol, and the negative control was high-density polyethylene (HDPE)^37^.

#### Thrombogenicity (Contact coagulation)

The thrombogenicity was measured using the intrinsic pathway^80^. The thrombogenicity of the biomaterials was evaluated using a whole blood kinetic clotting time method described elsewhere^80^. Briefly, the clotting reaction was activated by the addition of 850 µL of CaCl_2_ (0.1 M) to an 8.5 ml sample of citrated blood. A 100 µL volume of the activated blood was carefully placed on top of the biomaterials. All samples were incubated at 37 °C for 0, 5, 15, 25, 35 or 45 min. At each time point, the samples were incubated with 2.5 ml of distilled water for 5 min, and then a 100 µl sample was transferred to a 96-well plate with 100 µl of RD and allowed to react for 15 min at 37 °C. The erythrocytes that were not trapped in the thrombus were lysed with distilled water, thereby releasing haemoglobin into the water. The concentration of haemoglobin in solution was assessed by measuring the absorbance at 540 nm using a 96-well plate reader. HDPE was used as a reference material^49^, the size of the clot was inversely proportional to the concentration of haemoglobin in solution, and samples were tested in triplicate at three independent times.

To analyse plasma protein absorption, platelet-rich plasma (PRP) was extracted from whole blood at 300 g for 15 min. Then, the PRP was diluted by half with calcium and magnesium-free PBS, and 0.5 ml of the resulting solution was placed in contact with the material for 45 min at 37 °C. Finally, the materials were fixed and observed with a scanning electron microscope (SEM).

### Animal studies

Animal studies were carried out to understand the immune response performance of porous 3D biomaterials. The protocol was adapted from Seyednejad *et al*.^71^, Helenius *et al*.^30^ and Mendes *et al*.^31^. The detailed protocol is described below^30,31,71^.

#### Implantation protocol

The mice were housed together in a laboratory for animal studies at Corporación para Investigaciones Biologicas (CIB), Medellín, Colombia. Forty-eight Balb/c mice (Male, 6 weeks) were anaesthetized with 50 mg/kg ketamine and 2 wt. % of 10 mg/kg xylazine. A 1 cm dorsal incision was made on each mouse to create subcutaneous pockets. The biomaterials were transferred into the pocket of each mouse, and the skin was closed using Vicryl 4.0 sutures. As the control materials, HDPE and latex were used to elicit negative and positive reactions, respectively.

After 1, 4, 8 and 12 weeks, the animals were sacrificed using a CO_2_ chamber, and the biomaterials were extracted along with the surrounding tissues and were subjected to to a gross inspection along with the lymphatic nodes to look for signs of infection or necrotic tissues. Then, the biomaterials were fixed with 3.5 wt.% formaldehyde and processed into 4 groups (n = 2 per biomaterial per time point) to investigate either the tissue ingrowth into the scaffold micropores or the tissue reaction (immune response and collagen, elastin and cell infiltration) by histology.

#### Histological examination

The fixed biomaterials together with the ingrown tissues were dehydrated with a series of alcohol solutions and then transferred into xylene. The fixed biomaterials were subsequently embedded in paraffin. From the obtained paraffin blocks, three 5 µm thick sections were stained with haematoxylin-eosin (HE), Masson’s trichrome (MT) or Verhoeff-van Gieson (VVG).

The different samples were scored using light microscopy to look for signs of inflammation, cell infiltration, and neovascularization, among others. The observer scored the tissue morphology in 5 spots in both in the biomaterial periphery and its centre, as seen in Table 3, at 40X according to the ISO 10993-6 *“Biological evaluation of biomedical devices part 6-test for local effects after implantation”*. The percentage of tissue/cell infiltration was calculated using low magnification (10X) images according to Eq. 1. Areas were measured using ImageJ2^81^ and the brush area tool on calibrated images.

1 $$Percentage\,of\,tissue/cell\,in\,filtration=\frac{Infiltrated\,area\,(\mu {m}^{2})}{Total\,area\,(\mu {m}^{2})}\ast 100$$

**Table 3:** Criteria for microscopy evaluation of histology cuts according to ISO 10993-6 *“Biological evaluation of biomedical devices part 6-test for local effects after implantation”*.

Cell type/response	Score per high power (400X) field (phf)				
	0	1	2	3	4
Polymorphonuclear cells	0	Rare, 1–5/phf	5–10/phf	Heavy infiltrate	Packed
Lymphocytes	0	Rare, 1–5/phf	5–10/phf	Heavy infiltrate	Packed
Plasma Cells	0	Rare, 1–5/phf	5–10/phf	Heavy infiltrate	Packed
Macrophages	0	Rare, 1–5/phf	5–10/phf	Heavy infiltrate	Packed
Giant cells	0	Rare, 1–5/phf	5–10/phf	Heavy infiltrate	Packed
Necrosis	0	Minimal	Mild	Moderated	Severe
Neovascularization	0	Minimal capillary proliferation, focal, 1–3 buds	Groups of 4–7 capillaries with supporting fibroblastic structures	Broad band of capillaries with supporting structures	Extensive band of capillaries with supporting structures
Fibrosis	0	Narrow band	Moderately thick band	Thick band	Extensive band
Fatty Infiltrate	0	Minimal of fat associated with fibrosis	Several layers of fat and fibrosis	Elongated and broad accumulation of fat cells about the implant site	Extensive fat completely surrounding the implant
Collagen	0	Minimal presence	Mild presence	Moderated presence	Extensive presence
Elastin	0	Minimal presence	Mild presence	Moderated presence	Extensive presence
Traumatic necrosis	0	Minimal	Mild	Moderated	Severe
Foreign debris	0	Minimal	Mild	Moderated	Severe

### Microscopy

#### Scanning Electron Microscopy (SEM)

SEM was used to analyse the morphology of 2D and 3D BNC biomaterials, especially their interaction with erythrocytes and plasma proteins. For the observation of biomaterial morphology, the samples were frozen at −196 °C using liquid nitrogen and freeze-dried, and then they were coated with gold using an ion sputter coater. The specimens were observed with a JEOL JSM 5910 LV scanning electron microscope operating at 20 kV at 200X.

For the observation of the erythrocytes and plasma proteins, the samples were fixed in formaldehyde in PBS free of calcium and magnesium. Then, the samples were rinsed with ultrapure water to remove the salts, freeze-dried, coated and observed as previously explained. The magnification was 2000X.

#### Light microscopy

Histological cuts were observed using an upright Nikon microscope equipped with a DS-Fi3 integrated camera. Images were taken at 10X and 40X objectives using NIS-Elements basic research software (version 4.6; Laboratory imaging). ImageJ2 software^81^ was used to measure the distance of cell infiltration.

### Statistical analysis

Statistical analysis of the data was performed using one-way analysis of variance (ANOVA) using StatGraphics Centurion software (version 16.1.11; StatPoint Technologies Inc.). A p-value < 0.05 was considered statistically significant.

### Ethics statement

*Ex vivo* haemocompatibility assays needed the use of peripheral human blood, for this, two adult donors, in full awareness of the protocol, expressed their consent and sign the informed consent about the extraction of peripheral blood and the use of their blood in the present study. All experiments and the informed consent were approved by the Ethics Committee of the Universidad Pontificia Bolivariana, Medellín, Colombia in the approval letter emitted on 6^th^ of July of 2015. The experiments were carried out in accordance with the Declaration of Helsinki; the International Ethical Guidelines for Health-related Research Involving Humans (Council of International Organizations of the Medical Sciences and the World Health Organization); the law 23 of 1981, Colombia; and Resolution 008430 of October 4^th^, 1993 of the Ministry of Health of the Republic of Colombia.

Animal experimentation were in compliance with the Institutional Guidelines on the Use of Laboratory Animals of the Corporación para Investigaciones Biológicas (CIB), Medellín, Colombia. Moreover, the CIB ethics committee board approved all the animal experiments in the approval letter on 5^th^ of October of 2015. None of the committee board members emitted any negative vote.

The experiments were carried out in accordance with the European Community Guidelines for the Care and Use of Experimental Animals; the guide of care and use of laboratory animals stipulated in the public law 99–158 of 1985 “Animals In Research”, from the United States; and the scientific, technical and administrative standards established by legislation 008430 of 1993 and 2378 of 2008 of the Ministry of Health and Social Protection of Colombia.
